# Fetal Complex Congenital Heart Disease Diagnosed Using Prenatal Ultrasound and Corrosion Casting for Large Vessels: A Report for Authentic Teaching by True Representation

**DOI:** 10.4274/balkanmedj.galenos.2019.2019.6.13

**Published:** 2019-08-22

**Authors:** Peng An, Yu Wang, Li-min Xing, Wei Feng

**Affiliations:** 1 Department of Ultrasound, Xiangyang No. 1 People’s Hospital Affiliated to Hubei University of Medicine, Xiangyang Key Laboratory of Maternal-Fetal Medicine in Fetal Heart Disease, Hubei, China

To the Editor,

Complex congenital heart disease is the most common birth defect. According to the National Maternal and Child Health Care Surveillance report released by the Ministry of Health (China), the incidence of complex congenital heart disease is continually rising, with an annual increase of approximately 150,000-200,000 children. An accurate prenatal diagnosis of complex congenital heart disease is crucial to determine postnatal surgical plans (1).

Currently, prenatal ultrasound is the preferred method to diagnose fetal malformations and abnormalities; however, its accuracy in the diagnosis of complex congenital heart disease is not optimal. In particular, it is typically difficult to accurately show vascular travel and spatial adjacent relationships through ultrasound for certain conotruncal or aortic malformations, such as coarctation or interruption of the aortic arch, transposition of the great arteries, double outlet right ventricle, ectopic ductus arteriosus, and aberrant subclavian arteries (2,3). Diagnostic accuracy considerably depends on the experience and technique of the sonographer; therefore, using simulation to teach complex congenital heart disease diagnosis is extremely essential. However, current routine anatomy lectures can only well demonstrate complex congenital heart disease specimens initially because after immersion fixation, these specimens usually develop deformations, collapses, and damage and cannot accurately reproduce the vascular morphology of rare complex congenital heart disease. Notably, casting can display the true anatomical structure and space confirmation of complex congenital heart disease, thereby helping in teaching and clinical research purposes. Moreover, casting can help physicians to further understand fetal complex congenital heart disease and holds significant value in accurately diagnosing this type of disease before delivery. In addition, casting provides true morphological basis for prenatal diagnosis, teaching, research, and science popularization (4). Complex congenital heart disease is primarily characterized by malformations in the heart valves and vascular system, and corrosion casting of large vessels is advantageous in complex vascular malformations (5,6). We demonstrated the pathological results of large-vessel casts in one case of complex congenital heart disease, both of which was natural-death specimens after birth (infant death after intensive treatment and care). Dissection and casting were performed after obtaining approval by the ethics committee and signed informed consent from the parents and families.

A 30-year-old pregnant woman (gravida 1, para 0) at 23 + 5 weeks of gestation. Noninvasive prenatal test results indicated a trisomy 21, trisomy 18 and trisomy 13 were low risk. Prenatal ultrasound results revealed the coarctation of the aortic arch, double outlet right ventricle, and a small amount of the pleural and peritoneal fluid. The vascular cast exhibited double outlet right ventricle and complete transposition of the great arteries, interruption of the aortic arch (not aortic arch constriction), right ductus arteriosus, anomalous ductus arteriosus connections (left common carotid artery-ductus arteriosus-pulmonary artery), and aberrant left subclavian artery (from the descending aorta) and descending aorta starting from the beginning of the pulmonary artery (Figure 1).

**Modified vascular corrosion casting method:** 1- The specimen was washed with running water, and special attention was paid to ensure that the mouth and anus were cleaned, followed by disinfection with 10% bromo-geramine. 2- Cannulation: The abdominal wall was incised, the umbilical vein was dissected, a “V”-shaped small opening was made, and a cannula was introduced into the umbilical vein at the proximal end of the heart. The left umbilical artery was dissected and incised to facilitate the outflow of blood, clots, and the washout medium. 3- Lumen cleaning: The umbilical vein cannula was flushed with 20-50 mL of acetone to wash out the blood and clots and avoid their interference on the cast in the heart chambers and vascular lumina. 4- Infusion of casting medium: In total, 100-200 mL of acrylonitrile butadiene styrene resin was slowly infused into the specimen, which took approximately 30 minutes. 5- Corrosion: Twenty-four hours after the resin had cured, the specimen was immersed in 30% hydrochloric acid solution for acid etching. 6- Specimen rinse: After 7-10 days, the specimen was removed from the hydrochloric acid solution and carefully rinsed to get rid of the tissues. Then, the specimen was soaked in water to eliminate the residues.

**Note:** 1- Perfusion time: It is preferable to infuse within 24 hours to enhance the perfusion effect and prevent blockage from clotting in the vessels and heart chambers, which is generally caused by keeping the specimen for too long. 2- Casting medium concentration: When the concentration of the casting medium is too low, it is easy to infuse, but it shrinks too much during solidification and results in a thin and imperfect blood vessel cast. When the concentration is too high, it is difficult to infuse and results in a full cast but with a rough surface that can easily crack. Therefore, the optimal concentrations of the casting medium should be low (1%-3%) initially and then high (5%-8%), with initial fast infusion and subsequent slow infusion. 3- When air or clots are present in the heart chamber, the ventricle surface can be broken with a needle or cut to make a small incision while the casting medium is in a semi-solid state. After removing the air or clots, a small piece of gauze soaked in the casting medium is then filled into the heart chamber to recover its shape (6). 4- Selection of the appropriate cannula: The pediatric scalp needle is used. The needle is removed, and the tube is used as the cannula (Special reminders of limitations: The above perfusion methods are only suitable for specimens above 20 gestational weeks but not for specimens below 20 gestational weeks).

Notably, the feedback of the casting results is helpful in the further analysis and teaching of prenatal ultrasound imaging. Comparison between the casting results and prenatal ultrasound images could effortlessly explain several questionable images, thereby improving the accuracy of prenatal diagnosis. Echocardiography is highly accurate in the diagnosis of fetal intracardiac malformations, whereas the vascular casting technique is superior in displaying the complex vascular malformations of complex congenital heart disease and can be used to guide ultrasound diagnostic teaching.

## Figures and Tables

**Figure 1:**
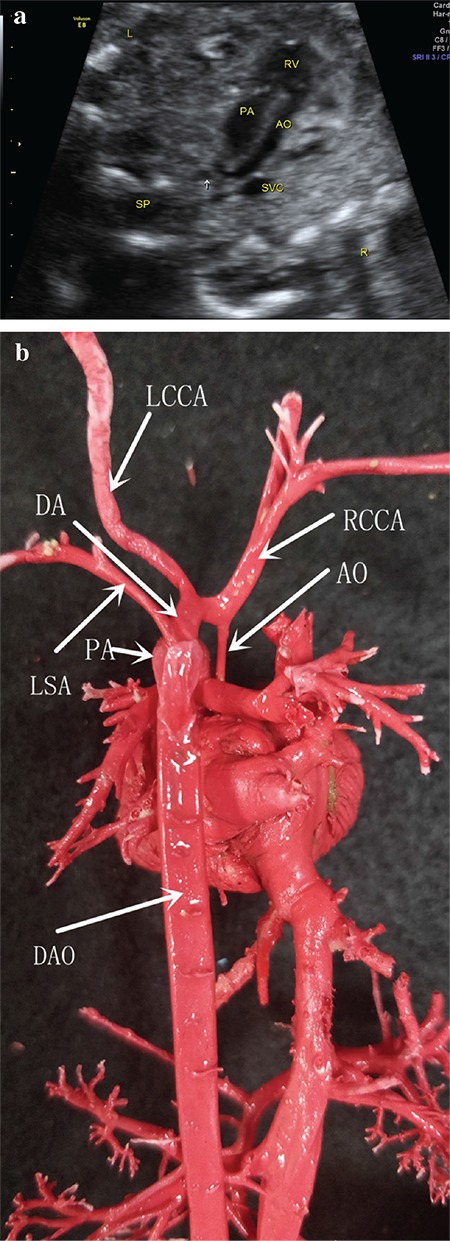
Prenatal ultrasound only diagnosed coarctation of the aortic arch and double outlet of right ventricle (a). The cast shows interruption of the aortic arch and ductus arteriosus abnormal connections (LCCA-DA-PA) (b). AO: Ascending aorta; DA: ductus arteriosus; DAO: descending aorta; LCCA: Left common carotid artery; LSA: Left subclavian artery; PA: pulmonary artery; RCCA: right common carotid artery

## References

[1] Yang XY, Li XF, Lu XD, Liu YL (2009). Incidence of congenital heart disease in Beijing, China. Chin Med J (Engl).

[2] van Velzen CL, Clur SA, Rijlaarsdam ME, Pajkrt E, Bax CJ, Hruda J, et al (2016). Prenatal diagnosis of congenital heart defects: accuracy and discrepancies in a multicenter cohort. Ultrasound Obstet Gynecol.

[3] Esmer AÇ, Yüksel A, Calı H, Ozsürmeli M, Omeroğlu RE, Kalelioğlu I, et al (2014). Prenatal diagnosis of persistent left superior vena cava and its clinical significance. Balkan Med J.

[4] Wang Y, Cao HY, Xie MX, He L, Han W, Hong L, et al (2016). Cardiovascular cast model fabrication and casting effectiveness evaluation in fetus with severe congenital heart disease or normal heart. J Huazhong Univ Sci Technolog Med Sci.

[5] Cao HY, Wang Y, Hong L, Han W, He L, Song BC, et al (2017). Morphological features of complex congenital cardiovascular anomalies in fetuses: as evaluated by cast models. J Huazhong Univ Sci Technolog Med Sci.

[6] An P, Liu W, Ning Y, Ye Y, Wang Y (2019). Type I Esophageal Atresia with Transposition of Great Arteries on Prenatal Ultrasound, Magnetic Resonance Imaging, and Vascular Cast. Balkan Med J.

